# In situ Fabrication of Multi-Walled Carbon Nanotubes/Silica Hybrid Colloidosomes by Pickering Emulsion Templating Using Trialkoxysilanes of Opposite Polarity

**DOI:** 10.3390/polym11091480

**Published:** 2019-09-10

**Authors:** Franziska Grzegorzewski, Avital Benhaim, Yafit Itzhaik Alkotzer, Einat Zelinger, Noga Yaakov, Guy Mechrez

**Affiliations:** 1 Department of Food Quality & Safety, Institute for Postharvest and Food Sciences, Agricultural Research Organization (ARO), Volcani Center, 68 HaMaccabim Road, Rishon LeZion 7505101, Israel; franziska@volcani.agri.gov.il (F.G.); avital.benhaim@gmail.com (A.B.); yafiti@volcani.agri.gov.il (Y.I.A.); nogay@volcani.agri.gov.il (N.Y.); 2 The Interdepartmental Equipment Unit, The Robert H. Smith Faculty of Agriculture, Food and Environment, The Hebrew University of Jerusalem, POB 12, Rehovot 7610001, Israel; einat.zelinger@mail.huji.ac.il

**Keywords:** Pickering emulsions, colloidosomes, microcapsules, multi-walled carbon nanotubes-silica nanocomposites

## Abstract

A simple and effective way to prepare multi-walled carbon nanotubes (MWNT)//silica hybrid microcapsules (colloidosomes) is presented. These microcapsules have been generated by emulsion templating in a biphasic oil-in-water (o/w) system. Two trialkoxysilanes of complementary polarity, (3-aminopropyl)triethoxysilane (APTES) and dodecyltriethoxysilane (DTES), were used to chemically immobilize the silica nanoparticles at the o/w interface and stabilize the as-generated Pickering emulsions. The effects of varying the o/w ratio and the concentration of the added solids on the type of emulsion formed, the oil droplet size, as well as the emulsion stability have been investigated. The emulsion phase fraction was dependent on the silica content while the droplet size increased with increasing oil volume percentage. A solid shell emerged around the oil droplets from copolymerization between silane monomers. The thickness of the resulting shells was several hundreds of nm. Although MWNTs and silica nanoparticles both were co-assembled at the o/w interface, silica has shown to be the sole stabilizer, with APTES being crucial for the formation of the shell structure. Drop-casting of the emulsion and air-drying led to hierarchical open porous MWNT-silica nanocomposites. These new structures are promising as electrically conductive thin films for variety of applications, such as electro-optics, encapsulation, or chemical sensing.

## 1. Introduction

The chemical inertness, thermal robustness, and the ability to easily introduce a diversity of functional groups on their surface make silica-based materials attractive for a broad range of applications [1,2]. However, they generally suffer from poor mechanical attributes such as brittleness and low resistance to mechanical stress, which greatly limits their use [3,4,5]. To bypass the above limitations silica-based materials are generally reinforced with fillers [6,7,8,9]. In this context, carbon nanotubes (CNT) are particularly promising [10,11,12,13,14]. This is mainly attributed to their outstanding mechanical, electrical, and optical properties [15,16,17], which are aimed to be imparted in the final composite. The incorporation of CNTs in a silica host matrix is thus an important technological step to tailor conductive silica-based superstructures and to explore novel applications in the field of optics or for electromagnetic interference shielding [18,19].

Unfortunately, the use of CNTs is limited by their poor dispersibility and low interfacial compatibility in most host matrices [20,21]. This is partly due to their strong agglomeration, driven by Van der Waals interactions. Consequently, successful approaches are trying to achieve a homogeneous dispersion and stronger interfacial interaction with the host matrix [22,23]. Examples for CNT/silica composites with various morphologies (such as films, powders, xerogels or fibers) have been widely studied [24,25,26,27,28,29,30].

In this study, we investigate the preparation of multi-walled CNT (MWNT)/silica microcapsules based on the self-assembly of silica and MWNTs at an o/w interface. These colloidal capsules, consisting of a particulate shell and a liquid core, are also called colloidosomes [31]. As they often exhibit properties different from the individual components that they are built of, they have gathered considerable attention in various fields such as catalysis, microencapsulation, controlled delivery or gas sensing [32,33,34,35,36]. Microcapsules and colloidosomes, in particular, are formed by means of various templating approaches [31,37,38,39,40,41,42,43,44], among which, emulsion templating is a particularly attractive method for the preparation of well-defined porous structures in many different materials [45,46]. Emulsion templating is based on the self-assembly of colloidal particles at the interface between two immiscible liquids, typically oil and water [47]. These structures are generally known as Pickering emulsions [48,49]. Particles with suitable surface chemistries do adsorb strongly to the liquid-liquid interface. The origin of their strong anchoring at the oil-water interface is the partial wetting of the particles’ surface by both liquids. The degree of wettability is expressed by the contact angle θ, where the liquid-vapor interface meets the solid-liquid interface. Besides wettability the adsorption of solid particles at the interface shows a clear particle size dependence. The energy barrier to re-migrate either to one of the two bulk phases is typically several orders of magnitude larger than the thermal energy, *k_B_*T [50,51,52]. The as-generated particle layer sterically hinders the close approach of the emulsion droplets, thereby stabilizing them against coalescence [53].

**Figure 1:**
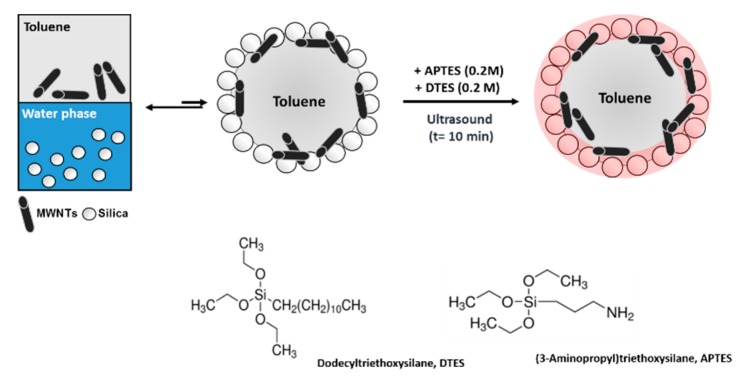
Schematic illustration of the formation of MWNT/silica microcapsules via particle-stabilized emulsion templating. The Pickering emulsion is stabilized by silica nanoparticles that assemble at the o/w interface; desorption is prevented by reacting silica with two silanes (APTES and DTES) of opposite solubility. MWNTs are co-assembling at the interface but will not act as stabilizers. Eventually, a core-shell structure is emerging from copolymerization of free and condensed silane monomers.

The literature of silica and CNT as Pickering emulsion stabilizers [54,55,56,57,58,59] and colloidosomes [42,43,60,61,62,63,64,65,66] is rich. However, to our best knowledge, this is the first time that a MWNT/silica microcapsule has been generated using a Pickering emulsion templating approach. An illustration of the methodology developed in this study is given in Figure 1.

To reduce the hydrophilicity of the fumed silica, the particles were functionalized in situ with two alkyltrialkoxysilanes of opposite polarity, (3-Aminopropyl)triethoxysilane (APTES) and Dodecyltriethoxysilane (DTES). This rendered the particles partially hydrophilic and partially hydrophobic and drove them to the o/w interface during the emulsification, where they were tightly immobilized. The MWNTs, preferentially accumulated at the o/w interface, without any functionalization, instead of being dispersed in any of the two bulk phases [67,68]. Interfacial polycondensation between hydrolyzed silane monomers produced almost instantaneously a polymeric siloxane layer at the interface in which the MWNTs were entrapped. Drop-casting and subsequent drying of the as-generated emulsions gave rise to hierarchical porous structures with various degrees of porosity.

This study presents a simple and effective route to prepare electrically conductive microcapsules of MWNTs embedded in a polysiloxane/silica matrix and to synthesize highly porous, reinforced ceramic films via emulsion templating, without the need for surfactants. These microcapsules could be used in a wide variety of applications, including, super capacitors and batteries, responsive slow reals systems and chemical and electrochemical sensors.

## 2. Materials and Methods

### 2.1. Chemicals and Buffers

MWNTs (NC7000TM, 95% purity) and carboxylated MWNTs (>8% carboxy functionalized) were obtained from Nanocyl SA (Sambreville, Belgium). Both have an average diameter of ~10 nm and an average length of 1.5 μm. Hydrophilic fumed silica (AEROSIL^®^ 300, 300 m^2^/g BET area, primary particle diameter ~7 nm, as provided by the manufacturer) was purchased from Evonik (Essen, Germany). (3-Aminopropyl)triethoxysilane (APTES, 99%), dodecyltriethoxysilane (DTES, technical grade), Nile Red (technical grade), 6-aminofluorescein (BioReagent), n-(3-dimethylaminopropyl)-n′-ethylcarbodiimide hydrochloride (EDC, BioXTra), and 2-(4-morpholino)ethanesulfonic acid hydrate (MES, ≥99.5%) and sodium dodecyl sulfate (SDS, BioReagent, ≥98.5%) were from Sigma-Aldrich (Steinheim, Germany). All chemicals were used without further purification. Toluene (analytical reagent grade) and water (Optima^®^LC/MS) were provided by Fisher Scientific Ltd. (Loughborough, UK).

### 2.2. Preparation of Silica Dispersions

Prior to emulsification, the as-received fumed silica nanoparticles (NPs) were suspended in ultrapure water by a high-intensity ultrasonic processor (Vibra-CellTM VCX 750, Sonics, USA) to give dispersions of 0.5, 1, 2, and 5 wt%. Sonication was done for 10 min using a 13-mm diameter probe tip, operating at 20 kHz with 750 W power and 35% amplitude. During sonication the vessel was cooled in an ice bath. The resulting dispersions were colorless or bluish in appearance. In all sonication processes described henceforth the same operating conditions were employed.

### 2.3. Pickering Emulsions by In Situ Functionalization of Silica

Emulsions were prepared by first dissolving MWNTs in a specific volume of toluene (1–5 mL). To these suspensions, 5–9 mL of the silica dispersions was added. The total volume of the as-generated biphasic systems was in all cases 10 mL. In this way, emulsions with different o/w ratios and varying amounts of MWNTs (1–5 mg) and silica NPs (0.5–5 wt%) have been prepared (Table 1). 500 μL of APTES and of DTES (both 0.2 M) were added to the biphasic mixtures and the systems were then emulsified using the same operating conditions as described above. The emulsions were then stored under ambient conditions until further analysis.

### 2.4. Preparation of Control Samples

Various control samples were prepared in order to determine the individual influence of each reactant on the oil droplet formation. To this end, the composition of each sample was varied, either including all components or leaving some of them out. All formulations were made of a 50:50 mixture of toluene and water (*v*/*v*). If not otherwise stated, the continuous aqueous phase consisted of 1 wt% of silica NPs. The amount of MWNTs (untreated and oxidized), if added, was 1 mg; in case of APTES and DTES, 500 μL of a 0.2 M stock solution were added. All mixtures were processed under identical conditions, as described above. The exact composition of each formulation is listed in Table 2.

### 2.5. Determination of Emulsion Stability and Emulsification Ability

The relative stability of the o/w emulsions was evaluated by determining the percentage of gravitational separation according to McClements [69]. To this end, the emulsions samples were gently agitated immediately after sonication to make sure they were initially homogeneous and then allowed to settle via gravitation/buoyancy. After a certain period (0.5, 6 d) the height of any distinct boundaries formed between different layers was then measured with a ruler. The extent of creaming can then be expressed by the so-called creaming index (*CI*) (%, Equation (1)) that is calculated as follows:

*CI* = (*H*_S_/*H*_E_) × 100 (1)

where *H*_S_ = total height of the transparent serum layer (here: water) at the bottom of the vials and *H*_E_ = total height of emulsion layer. An increase in the *CI* provides indirect information about the emulsion instability. In addition to the *CI*, the dispersed phase of the emulsion was measured and compared to the total phase volume of the mixtures. In this way, the emulsification ability of the mixtures has been investigated.

### 2.6. Bright Field Optical Microscopy

Image acquisition was done in bright field modus using an Olympus IX81 inverted microscope, equipped with a solid state laser with a 488 nm excitation laser line, and HC PL APO CS 20x/0.75 objective (with Leica Application Suite X software (LASX), Leica, Wetzlar, Germany). 1 mL of each emulsion was placed on a microscope slide and sealed with a cover slide in order to prevent evaporation of the solvents. Droplet size was analyzed using Fiji software [70,71] by measuring the droplet diameters from confocal microscopy images for each emulsion type. 

### 2.7. Fluorescence Labelling of MWNT Surface Functional Groups

**Figure 2:**
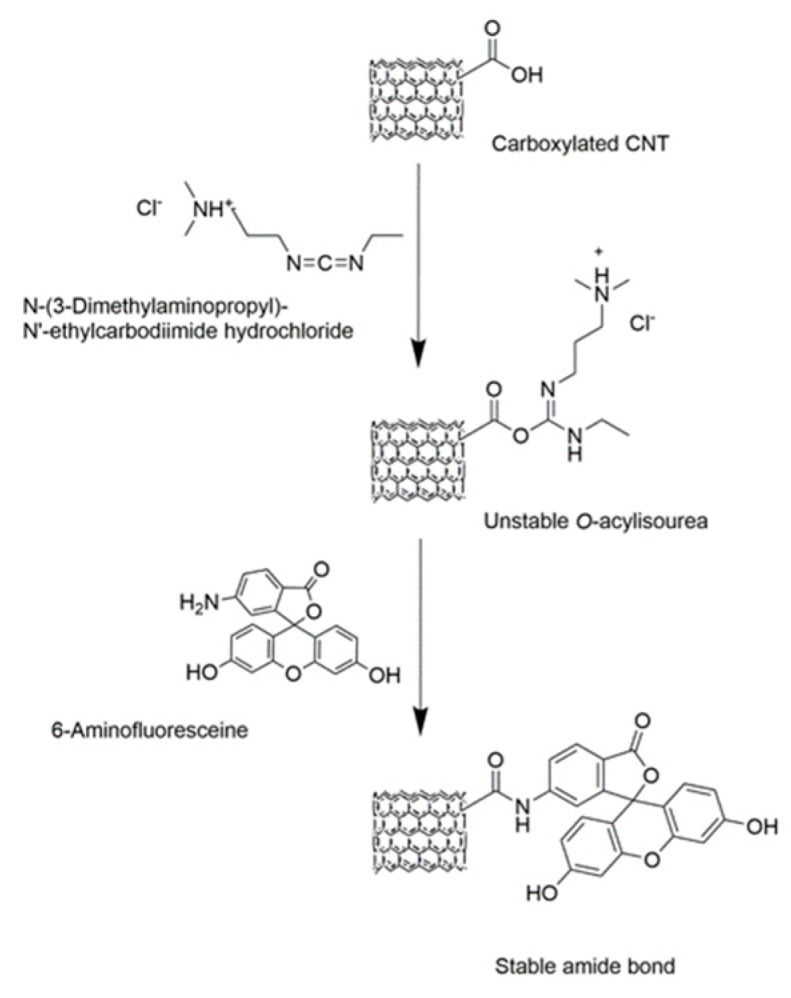
Fluorescence labelling of CNTs with 6-Aminofluorescein. Amidation of carboxylated CNTs with 6-AF proceeds in a two-step reaction using EDC as a cross-linker.

Carboxyl functionalized MWNTs were conjugated to 6-aminofluorescein (6-AF) via amidation reaction using N-(3-Dimethylaminopropyl)-N′-ethylcarbodiimide hydrochloride (EDC) as a zero-length cross-linker (Figure 2).

To this end, 50 mg of the MWNTs were dispersed in 10 mL of a 0.2 M SDS-solution and sonicated for two minutes at a 25% amplitude. Ten mL of the MWNT dispersion were then mixed with 30 mL EDC buffer solution (7 mM), 10 mL of buffered 6-AF solution (3 mM) and 60 mL of MES buffer (0.5 M). The reactants were vortexed for one hour under ambient conditions in dark environment. Solid products were recovered after precipitation in methanol and subsequent filtration. The obtained nanotubes were rinsed with methanol to remove unreacted excess dye in the solution and physisorbed 6-AF molecules. This was monitored by analyzing the remaining fluorescence of the supernatant (*λ*_exc_/_em_ = 488/520 nm) after centrifugation (20,379× *g* for 20 min, Sigma 3-18KS centrifuge from Sigma Laborzentrifugen GmbH, Germany) in a microplate reader (SynergyTM Neo2 with Gen5 2.0 Data Analysis Software, BioTek Instruments, Inc., Winooski, VT, USA). The fluorescent MWNTs were then dried overnight at 35 °C in a vacuum oven (Sheldon Manufacturing, Inc., OR, USA) and eventually used for the preparation of the emulsions. Prior to emulsification, 1 mL of Nile Red (0.03 mM, toluene) was added to stain the oil phase. Its distribution in the emulsion as well as the localization of the fluorescent MWNTs was followed by confocal laser scanning microscopy analysis.

### 2.8. Confocal Laser Scanning Microscopy (CLSM)

Confocal images were collected on a Leica SP8 confocal microscope (Leica Microsystems CMS GmbH, Wetzlar/Germany) equipped with an inverted microscope fitted with a 40× HC PL APO CS2 (1.10 NA) water immersion objective. Excitation of 6-AF and Nile Red was from the 488 nm and the 552 nm laser line of an OPS laser, respectively. The 1024 × 1024 images were collected using Leica Application Suite X software (Leica Microsystems CMS GmbH, Wetzlar/Germany).

### 2.9. High-Resolution Scanning Electron Microscopy (HR-SEM)

**Figure 3:**
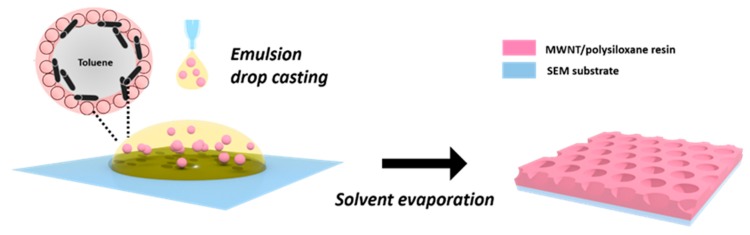
Preparation of MWNT/silica film structures. The MWNT/silica emulsion is drop-casted on a microscopic holder and dried for several hours at ambient conditions. After the solvent is evaporated, a solid composite structure is left, composed of a resinous polysiloxane-silica matrix in which MWNTs are individually embedded.

Measurements were performed using a MIRA3 field-emission SEM microscope (Tescan, Brno/Czech Republic) with an acceleration voltage of 7.0 kV and a secondary electron (SE) detector. Liquid samples were drop-casted on a conductive double stick carbon tape and dried at ambient conditions (Figure 3). Prior to imaging, a thin layer of iridium was evaporated onto the samples to render them electrically conductive, and avoiding surface charging by the electron beam.

### 2.10. Cryogenic-Field Emission Scanning Electron Microscopy

Cryogenic-field emission scanning electron microscopy (*cryo*-FESEM) analysis was performed on a JSM-7800F Schottky Field Emission Scanning Electron Microscope (Jeol Ltd., Tokyo/Japan). Liquid nitrogen was used in all heat exchange units of the cryogenic system (Quorum PP3010, Quorum Technologies Ltd., Laughton/United Kingdom). A small droplet of the freshly mixed emulsions was placed on the sample holder between two rivets, quickly frozen in liquid nitrogen for a few seconds and transferred to the preparation chamber where it was fractured (at −140 °C). The revealed fractured surface was sublimed at −90 °C for 10 min to eliminate any presence of condensed ice and then coated with platinum. The temperature of the sample was kept constant at −140 °C. Images were acquired with either a secondary electrons (SE), low electron detector (LED) or backscattered electron (BSE) detector at an accelerating voltage of 1 to 15 kV and a working distance of max. 10.1 mm.

### 2.11. Electrical Resistance Measurements

The electrical resistance of the MWNT/silica films was characterized by drop-casting the emulsions on silicon wafers on which an interdigital transducer (IDT) fabricated via standard photolithography was deposited. The silicon wafers were attached to a printed circuit board (PCB) that is connected to a dual-channel sensor management unit (±60 V, 3 A and ±200 V, 1A; Precision System PXI Source Measure Unit, National Instruments, USA). The electrical resistance of the resulting thin films was then measured after drying the films for 24 h at ambient conditions.

## 3. Results and Discussion

### 3.1. Preparation of MWNT/Silica Pickering Emulsions By In Situ Functionalization of Silica

**Figure 4:**
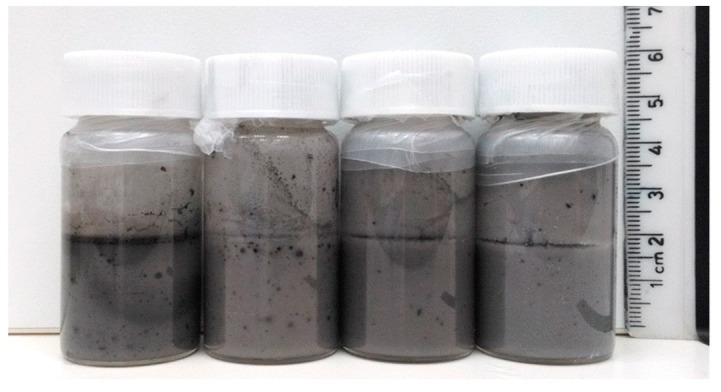
Toluene-in-water mixtures prepared from 2 wt% SiO_2_ and 1 mg MWNTs with 0.2 M APTES and DTES. The emulsions occasionally show the presence of MWNT aggregates, as illustrated by black dots within the otherwise typically homogeneous, milky-grey mixtures. From left to right: o/w ratio = 10:90, 20:80, 30:70, 50:50.

In total, 48 samples were prepared, subdivided in 3 × (4 × 4) groups (Table 1). Two types of experiments were carried out: First, the silica concentration was varied between 0.5 to 5 wt% while keeping the toluene volume fraction constant. Second, the toluene content was varied from 10–50 vol% at constant silica concentrations. These experiments were done for three different MWNT concentrations, ranging from 1 to 5 mg. The as-generated emulsions were milky-grey to dark black, depending on the amount of MWNTs used in the experiment. In some samples, the occurrence of black colored dots indicated the presence of agglomerated, not well dispersed MWNTs. Figure 4 shows an example of a series of emulsions immediately after emulsification, prepared with 1 mg MWNT in the toluene phase and 2 wt% silica in the aqueous phase, and toluene volume fractions ranging from 10 to 50 vol%.

**Figure 5:**
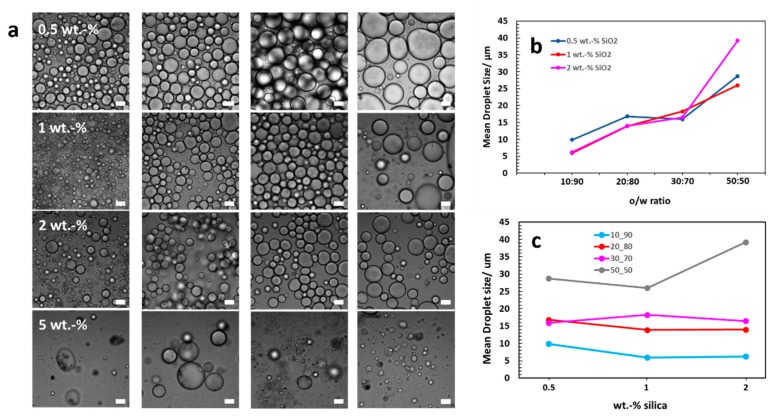
Mean droplet size analyzed by bright field microscopy for the 1 mg MWNT series. (**a**) Optical micrographs of MWNT/silica Pickering emulsions at different silica contents at o/w ratios. From left to right: o/w ratio = 10:90, 20:80, 30:70, 50:50. Scale bar is 20 μm. (**b**) The mean droplet size increases upon increasing oil vol% in the mixtures. Variances in between samples of different silica content were small and became significant just for 50:50 mixtures of the 2 wt% silica samples. (**c**) The average droplet diameter is independent of the amount of silica NPs used in the experiments. An increase in the droplet diameter only appears for a sample composition of 2 wt% silica and 50:50 o/w ratio. For simplicity reasons, only the successful 0.5–2 wt% silica series are shown.

**Figure A1:**
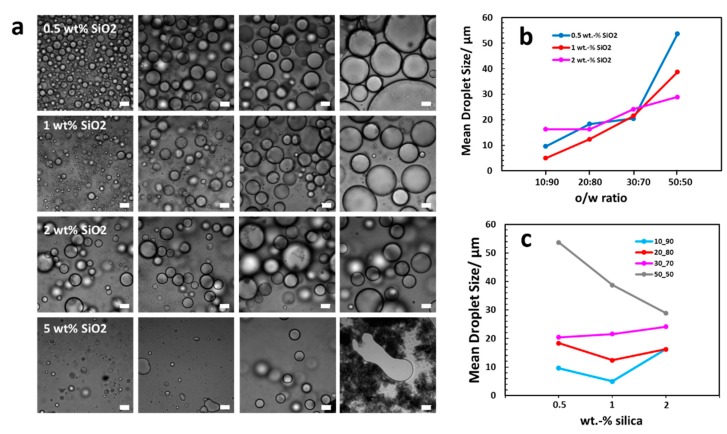
Mean droplet size analysed by bright field microscopy for the 2 mg MWNT series. (**a**) Optical micrographs of MWNT/silica Pickering emulsions at different silica contents at o/w ratios. From left to right: o/w ratio = 10:90, 20:80, 30:70, 50:50. Scale bar is 20 μm. (**b**) The mean droplet size increases upon increasing oil vol% in the mixtures. Variances in between samples of different silica content were small and became significant just for 50:50 mixtures. (**c**) Other than expected, the average droplet diameter did not decrease linearly with increasing silica content (exception: 50:50 mixtures). For a better understanding, only the successful 0.5–2 wt% silica series are shown.

**Figure A2:**
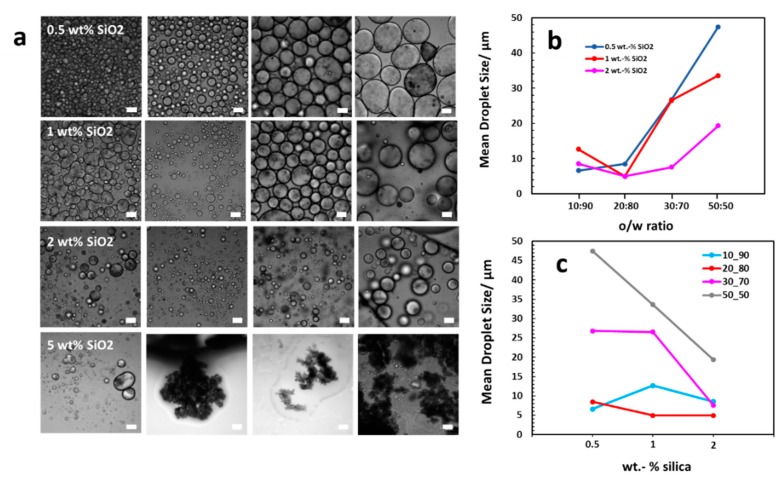
Mean droplet size analysed by bright field microscopy for the 5 mg MWNT series. (**a**) Optical micrographs of MWNT/silica Pickering emulsions at different silica contents at o/w ratios. From left to right: o/w ratio = 10:90, 20:80, 30:70, 50:50. Scale bar is 20 μm. (**b**) The mean droplet size grows upon increasing oil vol% in the mixtures. For low silica concentrations, the variances in the droplet diameters were small. Only at the highest o/w ratio, changes became substantial. 2 wt% silica samples showed an overall less steep increase. Droplet sizes stay more or less constant at around 10 μm until an o/w ratio of 30:70. Their size is doubled for the 50:50 o/w sample. (**c**) As for the other series in this study, also samples with a 5 mg MWNT content did not show any clear decrease in the droplet diameter when the silica content was increased (exception again: 50:50 mixtures). For a better understanding, only the successful 0.5–2 wt% silica series are shown.

In all of the cases, oil-in-water (o/w) emulsions were obtained. Success or failure of the emulsification depended mainly on the concentration of the silica NPs used in the experiments. Samples with 5 wt% silica concentrations barely emulsified; only samples with a 1 mg MWNT content revealed the generation of a small number of oil droplets under the microscope, within a thick silica water suspension. A reasonable analysis, however, was not possible due to the irregular shape and morphology of the very few emulsion droplets. Other than expected, the silica content had not an explicit influence on the droplet size in our experiments, although in general, a higher silica content should decrease the droplet diameter [72]. Figure 5 shows exemplarily the droplet size evaluation for the 1 mg MWNT series as observed in optical microscopy. Corresponding experimental data can be found in the Appendix A (Table A1). Our findings were inconsistent, as for example demonstrated in the 10 vol% oil ratio series (Figure 5a). Here, the droplet size decreases from 0.5 to 1 wt% silica content but then increased again for the 5 wt%. These features have been observed as well for the other series (Figure A1 and Figure A2 for 2 and 5 mg MWNT experiments, Appendix A). However, the droplet size shows a clear dependence on the o/w ratio and increases for all series (exception: the 5 wt% silica series) with increasing oil volume percentage (Figure 5b). Among the different silica contents, changes of the average droplet size at a given o/w ratio are generally small and became only significant at for the 50 vol% samples (Figure 5c). 

**Figure 6:**
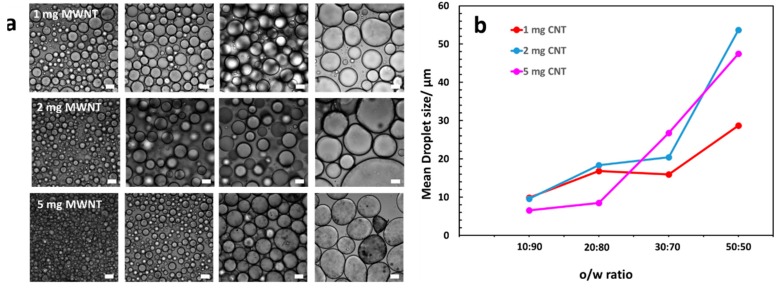
Influence of the MWNTs on the emulsion droplet size. (**a**) Bright field microscopy pictures for 0.5 wt% silica samples at various MWNT content. From left to right: o/w = 10:90, 20:80, 30:70, 50:50. Scale bar is 20 μm. (**b**) Despite small discrepancies, the mean droplet size is independent of the amount of MWNTs, but shows a strong dependence on the oil-volume fraction used in the individual emulsions. Significant differences only emerge at a 50:50 o/w ratio.

**Figure A3:**
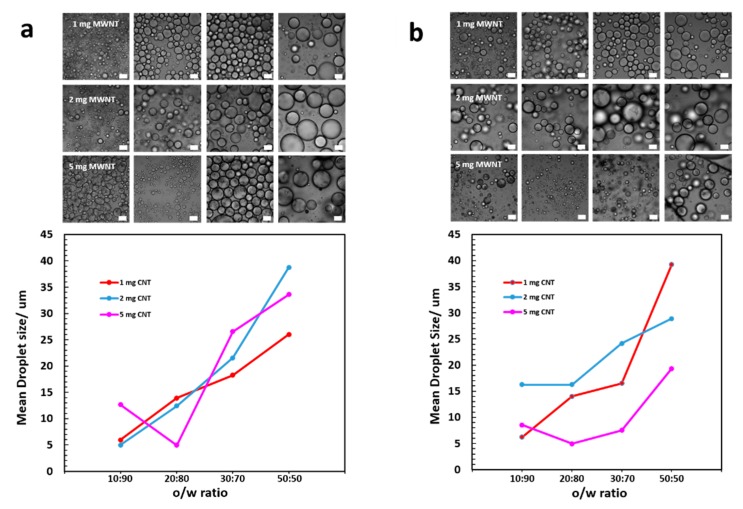
Influence of the MWNTs on the emulsion droplet size. Bright field microscopy images show the increase of the mean droplet diameter as a function of the o/w ratio for samples. Changes in between series with different MWNT contents become more obvious for samples with a higher silica content. Sample compositions: (**a**) 1 wt% silica, 1,2 and 5 mg MWNTs, 0.2 M APTES + DTES, (**b**) 2 wt% silica, 1, 2, and 5 mg MWNTs, 0.2 M APTES + DTES. From left to right: o/w = 10:90, 20:80, 30:70, 50:50. Scale bar is 20 μm. For better understanding, only the successful 0.5–2 wt% silica series are shown.

The amount of MWNTs did not show a substantial influence on the droplet size, as exemplarily demonstrated for the 0.5 wt% silica series in Figure 6. Figures for other silica contents can be found in the Appendix A (Figure A3). We therefore deduce, that silica is the only stabilizing particle in the system, and that the MWNTs are fixed at the droplet periphery without any substantial impact on the droplet stabilization process.

**Figure 7:**
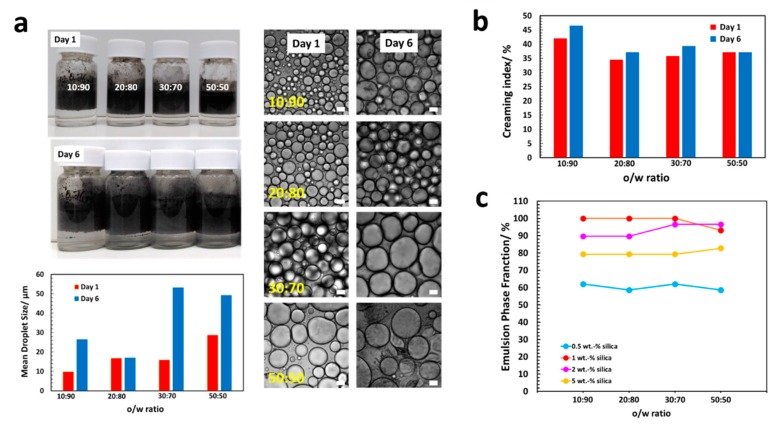
Determination of the emulsion stability of an o/w MWNT/silica Pickering emulsion. (**a**) Photographs and microimages of undisturbed emulsions immediately after emulsification and after one week of storage. Sample composition: 0.5 wt% SiO_2_, 1 mg MWNT. Except for the 20:80 sample, the oil droplet sizes increased with time due to coalescence of the emulsion droplets. Scale bar is 20 μm. (**b**) Creaming index of the same emulsions. Emulsions with a low silica content of 0.5 wt% creamed immediately (within 0.5 h) after emulsification. Except the 50 vol% sample, all emulsions undergo further creaming within six days. Changes, however, though are not substantial. (**c**) The emulsion phase fraction for emulsions stabilized by various concentrations of silica NPs as a function of the o/w ratio right after emulsification. The oil volume percentage did not show any substantial impact on the amount of toluene emulsified. Samples with a silica content of 0.5 or 5 wt% SiO_2_ were the least emulsified. At 1 wt% silica content, the full toluene was dispersed. Only at a 50:50 o/w ratio, the amount of silica NPs might be too low to ensure full emulsification. The amount of MWNTs in all samples was 1 mg.

Samples with a 0.5 wt% silica content and 1 mg MWNTs almost immediately formed a creamed layer after emulsification, coexisting with a clear supernatant aqueous phase at the bottom (Figure 7a, photographs). Over one week of storage, the emulsions underwent further creaming, though changes were not substantial (Figure 7b; droplet diameter values are listed in Table A2 in the Appendix A). Coalescence of the droplets could be clearly seen in optical microscopy: except for samples with a 20:80 ratio, the droplet size increased over time. Samples with a silica content higher than 0.5 wt% creamed slowly over 24 h after preparation but remained stable thereafter (data not shown). The emulsion volume fraction that formed immediately after emulsification is exemplarily shown for the 0.5 wt% silica samples in Figure 7c. Typically, the amount of the toluene dispersed was independent of the o/w ratio. Also, the amount of MWNTs in the mixture did not show any substantial impact. Samples with at 0.5 wt% silica content showed the smallest emulsion volume (60% with respect to the total volume of the mixtures). The largest emulsion volume was found for the 1 and 2 wt% silica samples, in which almost the total mixture was emulsified (~90–100%). 

**Figure A4:**
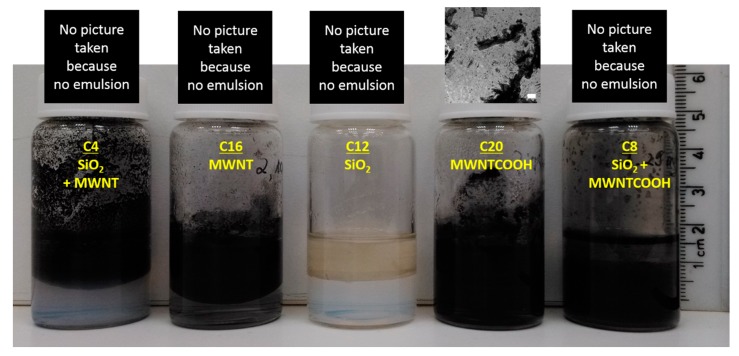
Toluene-water mixtures with different colloidal particles composition including bright field microscopy images. Samples were prepared without the addition of APTES and DTES. The toluene phase was 50 vol%. None of the studied systems emulsified. Phase separation occurred immediately after sonication. Scale bar is 20 μm.

In order to identify the key components, responsible for the successful emulsification, we prepared different control samples in which the individual composition was varied (Table 2). To rule out any surfactant effect stemming from the silane monomers in use, we first investigated the emulsification potential of an APTES/DTES o/w mixture without any additional stabilizers. Immediate phase separation took place right after sonication. Emulsions that were prepared with silica NPs only, without any addition of silanes and MWNTs, also phase-separated immediately after sonication (Figure A4). Obviously, the particles were completely wetted by the water phase and preferred to remain in the bulk water rather than staying fixed at the interface. Likewise MWNT-only mixtures (pristine and carboxylated) or the MWNT/silica mixtures showed an improved dispersion of the MWNTs in the oil phase (or water, in case of the carboxylated MWNTs) after the sonication, but no emulsion formed.

**Figure A5:**
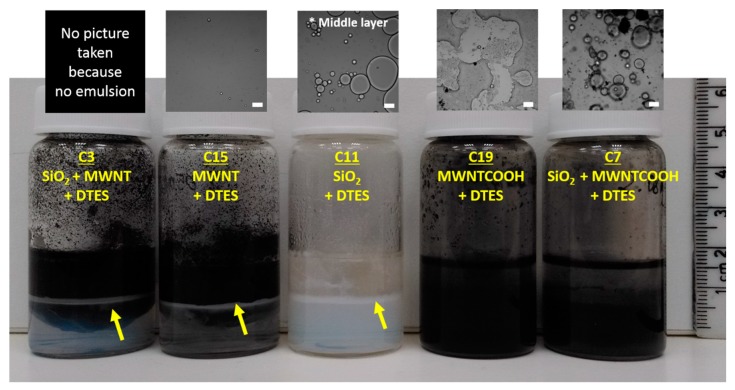
Toluene-water mixtures with different colloidal particles composition and addition of 0.2 M DTES, including bright field microscopy images. None of the studied systems visually emulsified. However, few emulsion droplets have been found for the MWNTCOOH/silica system. During storage, a whitish, turbid layer appeared in some samples (indicated by arrow). In all cases, the toluene phase was 50 vol%. Scale bar is 20 μm.

**Figure A6:**
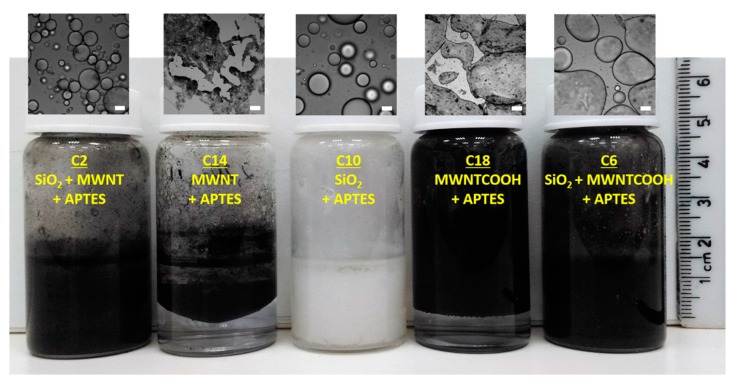
Toluene-water mixtures with different colloidal particles composition and addition of 0.2 M APTES, including bright field microscopy images. Samples that included silica all emulsified. The MWNT samples phase separated (pristine MWNTs, sample C14) or showed a Bijel-like structure (carboxylated MWNTs, C18). The toluene phase was 50 vol%. in all cases. Scale bar is 20 μm.

**Figure A7:**
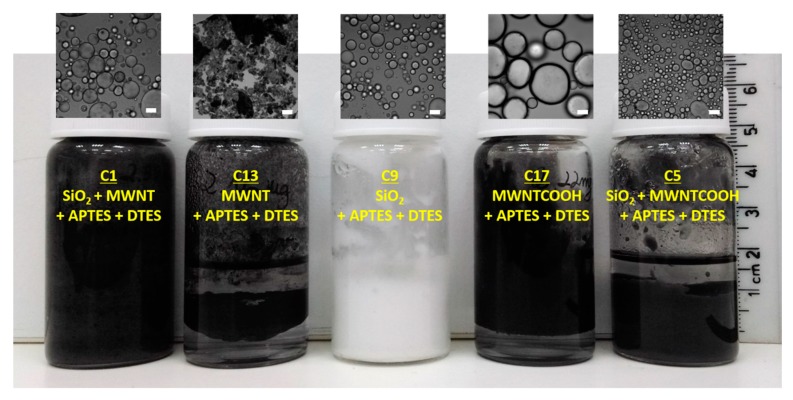
Toluene-water mixtures with different colloidal particles composition and addition of 0.2 M APTES and DTES, including bright field microscopy images. Samples that included silica all emulsified. Carboxylated MWNTs (sample C17) showed the formation of droplets. In case of C17, creaming occurs within the first 24 h, opposite to sample C5 where sedimentation could be observed. Pristine MWNT samples did not emulsify and phase separated immediately after sonication. The toluene phase was 50 vol%. in all cases. Scale bar is 20 μm.

The same behavior was observed when DTES was added to the mixtures (Figure A5). Although the functionalization of the silica NPs with DTES should turn silica particles more hydrophobic, any attempts to stabilize these emulsions failed. Most likely, the steric hindrance imposed by the bulky n-dodecyl side chain drastically decreases hydrolysis and polycondensation rates [73,74,75]. In addition, mixtures with MWNTs (pristine as well as carboxylated) did not emulsify and phase-separated immediately after sonication ended. DTES is thus neither reacting to a measurable extent with silica, but is also inert towards self-condensation. In case of the carboxylated MWNT/silica sample, analysis of the water phase showed the presence of few emulsion droplets. However, their number was minor and no visual emulsification could be observed. During storage, a whitish, turbid layer formed in some of the samples at the toluene-water interface. This layer did not appear in carboxylated MWNT samples. Its structural analysis is subject of current research. Things changed, when APTES instead of DTES was added to the mixtures (Figure A6). In this case, the silica NP mixtures easily reacted to homogeneous stable emulsions that showed similar microstructures than the original system, where both silanes were present in the mixture (Figure A7). Likewise, MWNT/silica samples easily emulsified, whereas the mixtures composed of MWNTs only remained phase-separated (pristine MWNTs) or showed the appearance of a narrow layer at the o/w interface with a Bijel-like structure (carboxylated MWNTs), together with a clear supernatant water phase at the vial bottom. 

From these finding, we therefore concluded, that two prerequisites need to be fulfilled in order to successfully generate a Pickering emulsion and eventually a MWNT/silica colloidosome core-shell structure: First, hydrophilic functional groups need to be present at the particle surfaces, such as the surface OH-groups at the silica surface. Without any reactive group at the particle surface, like in the case of the MWNTs, emulsification for systems like the ones presented here, will fail. Second, the choice of the silane monomer is crucial; the silanes need to be reactive towards hydrolysis and condensation in order to immobilize adequate particles at the o/w interface. In case they are prone to self-condensation, the oligomers that will be formed may further condense with Si-OH groups of the silica surface, resulting in a thick coating layer and eventually in a core-shell structure. 

### 3.2. Localization of MWNTs at the O/W Interface as Revealed by CLSM

**Figure 8:**
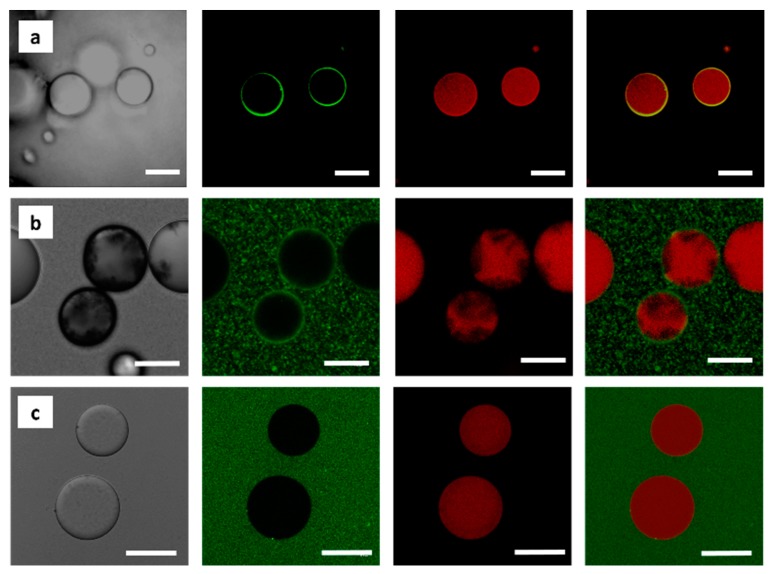
Visualization of the complex colloidal layer at the emulsion interface. (**a**) If MWNTs are fluorescently labelled with 6-AF, the oil droplets are surrounded by an intense green fluorescent layer indicating the presence of MWNTs at the o/w interface. The oil phase is easily identified by its intense red fluorescence, stemming from Nile Red. Sample composition: 20 vol% toluene-in-water with 2.0 wt% SiO_2_, 12 mg 6-AF conjugated MWNTs, and 0.2 M APTES and DTES. Scale bar is 20 μm. (**b**) In case of pristine MWNTs, the 6-AF cannot chemically conjugated to the nanotubes but is physisorbed to the MWNTs. Again, a green fluorescent layer is surrounding the droplet. This control experiment shows that also pristine MWNTs assemble at the o/w interface. Agglomerates of MWNTs are visible inside the oil droplets. Some of the MWNTs. However, were also transferred into the water phase, where an intensive aggregation was observed due to their inherent hydrophobicity. Sample composition: 50 vol% toluene-in-water with 2.0 wt% SiO_2_, 1 mg MWNTs, and 0.2 M APTES and DTES. Scale bar is 10 μm. (**c**) In the absence of MWNTs in the silica emulsions, no green fluorescent layer at the droplet periphery can be seen. 6-AF remains solved in the water phase without any adsorption to the silica particles. Control system made of 50 vol% toluene-in-water with 2.0 wt% SiO_2_, and 0.2 M APTES and DTES. Scale bar is 50 μm. From left to right: Bright field image, confocal images (green and red channels), and overlaid channels.

To visibly locate the MWNTs at the o/w interface of the emulsions, we conjugated the fluorophore 6-aminofluorescein (6-AF, *λ*_exc_/_em_ = 488/525 nm) to the free COOH- groups of carboxyl functionalized MWNTs. We also added the lipid staining dye Nile red during emulsification to confirm the presence of an o/w emulsion. The locus of the MWNTs at the o/w interface was then observed by CLSM. The occurrence of green fluorescent rings, constituting the periphery of the emulsion drop, demonstrated that the MWNTs are indeed assembled mostly at the droplet interfaces (Figure 8a). As expected, the inside of the droplet was red colored, clearly evidencing the presence of an o/w emulsion. While the covalent binding of particles to a chromophore is a common technique in fluorescence imaging to reveal their locus in a specific system, 6-AF-functionalized MWNTs might, however, show increased hydrophilicity as compared to the non-functionalized MWNTs that were used throughout all other experiments. Furthermore carboxylated MWNTs may be greatly shortened in their tube length due to the harsh acidic preparation process [76]. To rule out, that these changes affect the solubility of the MWNTs and their localization in the system, we analyzed the behavior of pristine MWNTs by CLSM. In addition, we compared our findings to a control sample, in which MWNTs were completely absent and the Pickering emulsions stabilized by silica particles only. In these control experiments, a covalent binding of 6-AF was not possible, so that 6-AF and Nile Red were simply added to the corresponding mixtures prior to emulsification. 

In case of the pristine MWNT mixture, an identical green circular layer surrounding the oil droplet was visible (Figure 8b). Besides, large green clusters could be seen in the continuous water phase. We believe that these two phenomena resulted from physisorbed 6-AF molecules at the MWNTs surface, likely via π- π stacking and Van der Waals interactions [77]. If so, the presence of non-fluorescent MWNTs inside the oil droplet would suggest, that the shell is not permeable to any molecular mass transfer. As expected for the silica system, only the major water phase was homogenously green colored, but green ring structures completely missing (Figure 8c).

### 3.3. Morphology and Shell Thickness of the MWNT/Silica Microcapsules

**Figure 9:**
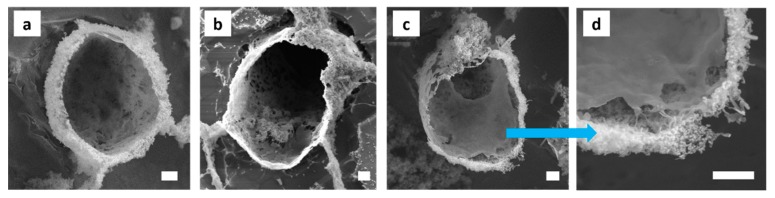
Cryo-SEM micrographs of the MWNT/silica emulsions. (**a**–**d**) Silica NPs are located at the interface and in the aqueous continuous phase. A polymeric smooth layer is formed at the inner side of the capsules with MWNTs embedded in between outer and inner shell layers. Sample composition: 10 vol% toluene-in-water with 2 wt% SiO_2_, 1 mg MWNTs, and 0.2 M APTES and DTES. Scale bar is 1 μm.

**Figure A8:**
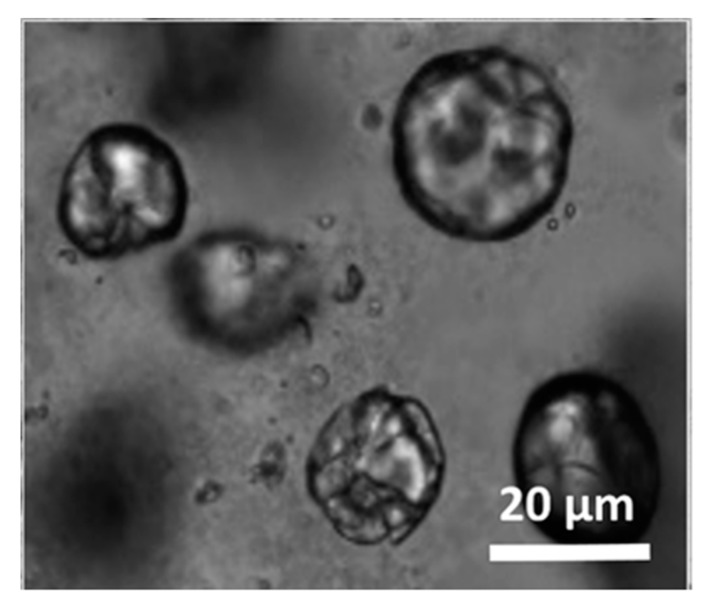
Bright field microscopy image showing micron-sized crumpled shell structures. Crumpling occurs when the volatile toluene is evaporating upon drying (sample composition: 10 vol% toluene-in-water, 1 wt% SiO_2_, 1 mg MWNTs, and 0.2 M APTES and DTES). Images of the emulsion droplets were recorded using an EVOS^®^ FL Auto microscope equipped with a high-sensitivity interline CCD camera and assisted with EVOS^®^ FL Auto software (life technologies, Carlsbad, CA/USA).

As evaporation of the volatile toluene proceeded during optical microscopy imaging, the droplets showed pronounced buckling and crumpling. This indicated that the oil droplets were encapsulated by a thin shell (Figure A8). The morphology and thickness of the shells were thereupon determined by cryogenic field-emission SEM. The microcapsules did not appear in all cases to be perfectly spherical and some rather showed an ellipsoidal shape (Figure 9). Their sizes ranged from 6 to 10 μm, which fits to the droplet sizes analyzed by optical microscopy.

**Figure 10:**
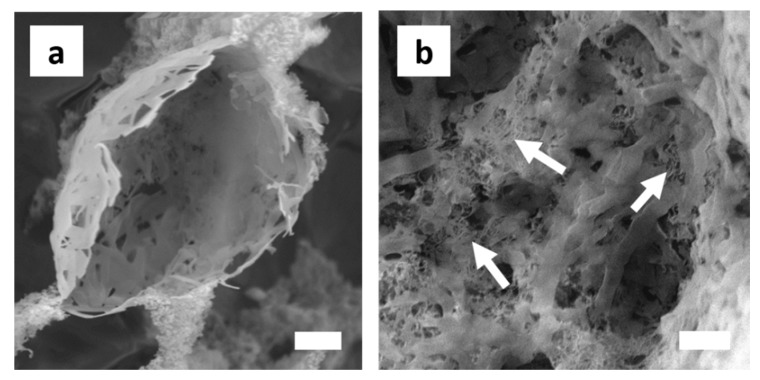
Cryo-SEM micrographs of ribbon-like polymeric structures. (**a**) The structures are randomly branched and of varying width and length. They probably form upon copolymerization of hydrolysed silane monomers that remained unreacted. Sample composition: 10 vol% toluene-in-water with 2 wt% SiO_2_, 2 mg MWNTs and 0.2 M APTES and DTES; (**b**) MWNTs, here indicated exemplarily by arrows, are embedded within the polymer matrix. Sample composition: 20 vol% toluene-in-water with 2 wt% SiO_2_, 2 mg MWNTs, and 0.2 M APTES and DTES. Scale bar in both images is 1 μm.

The shell forming layer was several hundreds of nanometers thick. The outer shell was composed of ‘amorphous’ silica NP aggregates. A smooth polymeric layer responsible for the shell formation can be clearly observed at the inner side of the capsules, especially illustrated in Figure 9c,d. In between these two layers, MWNTs could be observed. Most noticeable, however, were ribbon-like structures of several hundreds of nanometer thickness and varying length and ramification that formed at the o/w interface of some droplets (Figure 10). Close inspection shows that the MWNTs are fully incorporated into the polymer matrix. 

### 3.4. Highly Interconnected Porous Structures Form in Solid State

**Figure 11:**
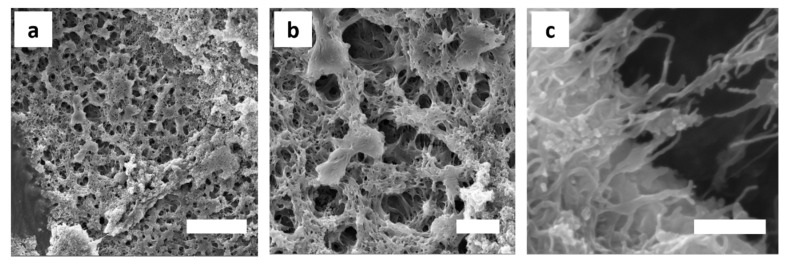
Micro- and nanostructures of the MWNT/silica films. (**a**,**b**) Drying of the emulsions generates solids with a complex, hierarchical architecture of open porosity and highly interconnected hollow spherical compartments of non-uniform size. Sample composition: 10 vol% toluene-in-water with 1.0 wt% SiO_2_, 1 mg MWNTs, and 0.2 M APTES and DTES. Scale bar is 10 μm (**a**) and 2 μm (**b**). (**c**) Silica particle decorated MWNTs are forming the skeleton. They are embedded within a polymeric matrix that forms upon copolymerization of the silane monomers and potential oligomeric side products. Sample composition: 50 vol% toluene-in-water with 0.5 wt% SiO_2_, 1 mg MWNTs, and 0.2 M APTES and DTES. Scale bar is 500 nm.

The ability of MWNTs to directly improve the mechanical and electrical properties of composite materials, is closely coupled to their uniform and individual dispersion within the host matrix. MWNT agglomerates that are caused by a Van der Waals interaction and intense MWNT entangling throughout ceramic or polymeric matrices are thus major barriers to success [78]. We therefore characterized the structure of the MWNT/silica emulsions by HR-SEM in order to analyze the dispersibility and the interfacial compatibility of the MWNTs in the final solid nanocomposite. Under the severe high vacuum conditions in the SEM chamber, the shells ruptured and collapsed. This indicated that the pure scaffold is still too feeble to persist to the mechanical forces exerted upon drying and to maintain the microcapsules shape. SEM analysis however showed a hierarchical, highly open porous network structure, with pores of 1-2 μm in diameter (Figure 11).

Smaller, secondary pores of few hundreds of nm were evident in the pore walls, interconnecting the neighboring larger cavities and showing potential for convective mass transfer. These pore throats probably resulted from the comparably thin polymeric layer that was formed between the droplets. Due to the density differences between the polysiloxane network and the aqueous continuous phase, shrinkage occurs causing ruptures in the polymer film at its thinnest point formed due to the stress exerted during the vacuum drying process [79]. Cavities and pores throats were irregularly shaped. In some cases, the morphology resembled a fibrillary mesh. Well-dispersed and individualized MWNTs could be clearly seen throughout the polymer pore walls. They were partly decorated by silica primary particles agglomerates (Figure 11c,d).

### 3.5. Electrical Resistance Measurements of the MWNT/Silica Films

**Figure 12:**
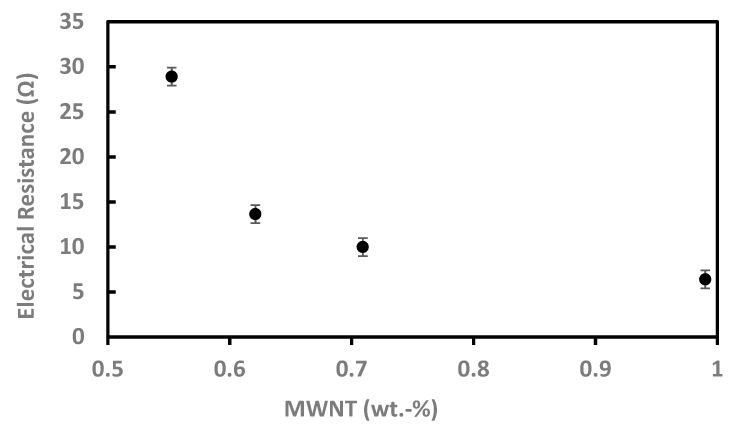
Electrical resistance of the MWNT/silica films vs. the MWNT content. Increasing the MWNT amount reduced the electrical resistance of the films.

Figure 12 shows the electrical resistance of the resulting films as a function of the MWNT content. The experiments were performed for four samples and with 9 cycles at zero pressure and room temperature. An electrical resistance of 29 Ω was obtained at a MWNT content of 0.55 wt%. This value is relatively low and indicates that the resulting films are electrically insulating. As expected, the electrical resistance decreased by 77% to a value of 6.5 Ω by increasing the MWNT content to 0.99 wt%. Our results demonstrate that porous conductive nanocomposite films can be fabricated following the methodology developed in this study. By altering the MWNT loading in a larger range, further fine tuning of the electrical resistance is expected. 

## 4. Conclusions

A simple strategy for the fabrication of MWNT/silica colloidosomes has been demonstrated. To the best of our knowledge, this is the first time that a hybrid CNT/silica microcapsule is prepared. Our approach is based on the self-assembly of silica particles and MWNTs at the o/w interface. The as-generated Pickering emulsion is stabilized by two trialkoxysilanes of opposite polarity that chemically react with the silica from the aqueous as well as from the organic phase and thus immobilize the silica nanoparticles at the interface. Despite the fact that the MWNTs are co- assembled at the o/w interface with silica particles, silica seems to be the sole stabilizing particle in our system. A shell is formed in situ via copolymerization of excess hydrolyzed silanes, with the MWNTs incorporated in the as-generated silica-siloxane network. To generate the siloxane layer, APTES has turned out to be the key silane. However, due to the numerous complex side reactions of and between the silane monomers, the exact formation mechanism of the siloxane shell has not yet been decisively clarified and is currently the subject of further studies. Drop-casting and air-drying of the emulsions left electrically conductive, porous MWNT/silica composites with 3D hierarchical open architectures. The individual dispersion of the MWNTs in the emulsion and their patterned arrangement in the composite film make these materials promising candidates for applications in the field of sensors, coatings, encapsulation or nanoelectronics. Further investigations will reveal the influence of nanoparticles with varying size, shape, and hydrophobicity to allow control of the droplet size in the emulsion template and hence of the structure in the resulting porous solid films. 

## Figures and Tables

**Table 1:** Composition of the individual MWNT/silica toluene-in-water emulsions used in this study. Samples were prepared for four different silica contents, and three different MWNT concentrations. The oil volume in the mixtures ranged from 10–50 vol%.

	wt% SiO_2_	vol% Toluene			
1 mg MWNT	0.5	10	20	30	50
	1	10	20	30	50
	2	10	20	30	50
	5	10	20	30	50
2 mg MWNT	0.5	10	20	30	50
	1	10	20	30	50
	2	10	20	30	50
	5	10	20	30	50
5 mg MWNT	0.5	10	20	30	50
	1	10	20	30	50
	2	10	20	30	50
	5	10	20	30	50

**Table 2:** Composition of the control samples. The amount of the particles, when added to the mixtures, was kept constant in all experiments, and consisted of 1 mg CNTs and/or 1 wt% silica, respectively. All samples were emulsified for 10 min and immediately analyzed.

Emulsion No.	Particle 1	Particle 2	Silane 1	Silane 2
C1	SiO_2_	MWNT	APTES	DTES
C2	SiO_2_	MWNT	APTES	-
C3	SiO_2_	MWNT	-	DTES
C4	SiO_2_	MWNT	-	-
C5	SiO_2_	MWNT-COOH	APTES	DTES
C6	SiO_2_	MWNT-COOH	APTES	-
C7	SiO_2_	MWNT-COOH	-	DTES
C8	SiO_2_	MWNT-COOH	-	-
C9	SiO_2_	-	APTES	DTES
C10	SiO_2_	-	APTES	-
C11	SiO_2_	-	-	DTES
C12	SiO_2_	-	-	-
C13	-	MWNT	APTES	DTES
C14	-	MWNT	APTES	-
C15	-	MWNT	-	DTES
C16	-	MWNT	-	-
C17	-	MWNT-COOH	APTES	DTES
C18	-	MWNT-COOH	APTES	-
C19	-	MWNT-COOH	-	DTES
C20	-	MWNT-COOH	-	-
C21	-	-	APTES	DTES

## Appendix A

**Table A1:** Average droplet diameters for the MWNT/silica toluene-in-water emulsions used in this study. The partially high standard deviation originates from the polydisperse droplet size distributions in some samples.

	wt% SiO_2_	Mean Droplet Diameter (±Standard Deviation)/μm			
		10 vol% Toluene	20 vol% Toluene	30 vol% Toluene	50 vol% Toluene
1 mg MWNT	0.5	9.9 (±6.4)	16.8 (±8.6)	15.9 (±13.0)	28.7 (±20.1)
	1	5.9 (±2.7)	13.9 (±6.5)	18.3 (±6.3)	26.0 (±14.0)
	2	6.2 (±4.4)	14.0 (±3.4)	16.5 (±6.7)	39.2 (±22.0)
	5	21.7 (±24.5)	46.8 (±38.0)	10.9 (±10.5)	17.6 (±10.2)
2 mg MWNT	0.5	9.6 (±4.5)	18.3 (±9.8)	20.5 (±11.7)	53.7 (±17.5)
	1	5.0 (±2.9)	12.4 (±6.7)	21.6 (±9.7)	38.7 (±23.5)
	2	15.5 (±11.5)	16.3 (±9.1)	24.1 (±12.5)	28.9 (±10.6)
	5	6.0 (±4.4)	8.3 (±5.4)	42.8(±25.0)	16.4 (±7.1)
5 mg MWNT	0.5	6. 6 (±2.3)	8.5 (±3.8)	26. 8 (±9.8)	47.5 (±11.3)
	1	12.7 (±6.3)	4.9 (±2.5)	26.5 (±8.3)	33.6 (±21.0)
	2	8.5 (±8.0)	4.9 (±3.0)	7.5 (±3.6)	19.3 (±10.0)
	5	nd	nd	nd	nd

nd—not determinable.

**Table A2:** Average droplet diameter changes of freshly prepared and one week old MWNT/silica toluene-in-water emulsions used in this study. Sample composition: 0.5 wt% silica, 1 mg MWNT, 02 M APTES and DTES. The partially high standard deviation originates from the polydisperse droplet size distributions in some samples.

	Vol% Toluene	Mean Droplet Diameter (±Standard Deviation)/μm	
		Day 1	Day 2
**1 mg MWNT** **0.5 wt% silica**	10	9.9 (±6.4)	26.5 (±12.4)
	20	16.8 (±8.6)	17.1 (±10.8)
	30	15.9 (±13.0)	53.3 (±11.3)
	50	28.7 (±20.1)	49.3 (±20.3)
